# Beta-blockade in V-V ECMO

**DOI:** 10.1186/s13054-024-04923-1

**Published:** 2024-04-25

**Authors:** Aravind K. Bommiasamy, Bishoy Zakhary, Ran Ran

**Affiliations:** 1 Division of Pulmonary and Critical Care Medicine, Oregon Health and Science University, Portland, OR USA; 2 Department of Emergency Medicine Critical Care Section, Oregon Health and Science University, Portland, OR USA

To the Editor,

We read with great interest Staudacher et al.’s illustrative cases of beta-blocker therapy on V-V ECMO [1]. The authors’ excellent work reminds us not to focus on the easily measured arterial oxygen saturation (${S}_{a}{O}_{2}$) but rather on the much more physiologically important variable of delivered oxygen (${DO}_{2}$). Herein, we demonstrate physiologically and mathematically that beta-blockade for a patient completely dependent on V-V ECMO will always decrease ${DO}_{2}$ irrespective of its effect on ${S}_{a}{O}_{2}$.

To illustrate this concept mathematically, there are some reasonable assumptions that must be made. The first is that ECMO effective blood flow rate (EF) are within normal operational parameters of the membrane lung and remain constant during beta-blockade. Second, that the membrane lung is well functioning such that the post-membrane lung blood oxygen saturation (${S}_{m}{{\text{O}}}_{2}$) is 100%. Third, that the patient’s lungs are non-functional and contribute no oxygenation to the blood. Finally, given the relatively small contribution of dissolved oxygen to total oxygen content, we ignore 0.03 × ${P}_{m}{O}_{2}$ in the calculation to simplify the math. With these assumptions in place, the arterial saturation on V-V ECMO equation simplifies to Eq. (1):

1 $$\begin{gathered} S_{a} O_{2} = \frac{EF}{{CO}} \times S_{m} O_{2} + S_{v} O_{2} \left( {1 - \frac{EF}{{CO}}} \right) + 0.03 \times P_{m} O_{2} \hfill \\ S_{a} O_{2} = \frac{EF}{{CO}} + S_{v} O_{2} \left( {1 - \frac{EF}{{CO}}} \right) \hfill \\ \end{gathered}$$

Equation 1: Patient arterial oxygen saturation on V-V ECMO simplified

If we rewrite Eq. (1), we get Eq. (2) [2]:

2 $$S_{a} O_{2} = \frac{{EF + S_{v} O_{2} \left( {CO - EF} \right)}}{CO}$$

Equation 2: Patient arterial oxygen saturation rewritten

Likewise, if we rewrite the Fick equation, we get Eq. (3):

3 $$S_{a} O_{2} = S_{v} O_{2} + \frac{{VO_{2} }}{13.4 \times Hgb \times CO}$$

Equation 3: Fick’s Equation solved for ${S}_{a}{O}_{2}$

Equating Eqs. (2) and (3), then solving for S_v_O_2_, we get Eq. (4):

4 $$S_{v} O_{2} = 1 - \frac{{VO_{2} }}{13.4 \times Hgb \times EF}$$

Equation 4: Determinants of ${S}_{v}{O}_{2}$

Therefore, we find that though ${S}_{v}{O}_{2}$ is traditionally dependent on *CO*, this is not necessarily true on V-V ECMO. Its covariates, *CO* and ${S}_{a}{O}_{2}$, cancel out in the idealized scenario proposed above. The only determinants of ${S}_{v}{O}_{2}$ on V-V ECMO, then, are ${VO}_{2}$, Hgb, and EF as seen in Eq. (4).

Finally, we explore the effect of beta-blockers on delivered oxygen (${DO}_{2}$) with the specific question, does the increase in ${S}_{a}{O}_{2}$ triumph over the reduction in *CO* or *vis versa*?

Simplifying the DO_2_ equation:

5 $$DO_{2} = 13.4 \times Hgb \times CO \times S_{a} O_{2}$$

Equation 5: Oxygen delivery simplified

Combining Eq. (3) with Eq. (5):

6 $$\begin{gathered} DO_{2} = 13.4 \times Hgb \times CO \times \left( {S_{v} O_{2} + \frac{{VO_{2} }}{13.4 \times Hgb \times CO}} \right) \hfill \\ \quad \quad \; = 13.4 \times Hgb \times CO \times S_{v} O_{2} + VO_{2} \hfill \\ \end{gathered}$$

Equation 6: ${DO}_{2}$ with relation to ${S}_{v}{O}_{2}$

Lastly, combining Eq. (6) with Eq. (4):

7 $$DO_{2} = 13.4 \times Hgb \times CO \times \left( {1 - \frac{{VO_{2} }}{13.4 \times Hgb \times EF}} \right) + VO_{2}$$

Equation 7: ${DO}_{2}$ on V-V ECMO expressed independent of ${S}_{a}{O}_{2}$ and ${S}_{v}{O}_{2}$

This final equation expresses delivery of oxygen on V-V ECMO as dependent only on hemoglobin, cardiac output, ECMO effective blood flow rate, and the body’s consumption of oxygen. Introducing beta-blocker therapy, then, irrespective of its effect on ${S}_{a}{O}_{2}$, reduces delivery of oxygen through reduction of cardiac output if Hgb, EF, and ${VO}_{2}$ remains constant. While it is conceivable that beta-blocker therapy could reduce ${VO}_{2}$ by decreasing myocyte oxygen consumption, thereby increasing ${DO}_{2}$, this effect is unlikely in normal physiologic ranges because myocyte oxygen consumption is typically only 10% of total body oxygen consumption (6–8 ml/100 g/min). At maximal inotropy and chronotropy, however, myocyte oxygen consumption could become a nontrivial factor and beta-blocker therapy may have utility as an antihypertensive agent in the tachycardic patient. For the vast majority of cases, however, beta blockade is not indicated during V-V ECMO as it effectively reduces DO_2_.

## Data Availability

Not applicable.

## Abbreviations

V-V ECMO: Veno-venous extracorporeal membrane oxygenation
$DO_{2}$: Patient oxygen delivery
$S_{a} O_{2}$: Patient arterial oxygen saturation
$EF$: ECMO flow rate
$S_{m} O_{2}$: Post-oxygenator oxygen saturation
$VO_{2}$: Patient oxygen consumption
$$Hgb$$: Hemoglobin
$P_{m} O_{2}$: Partial pressure of oxygen post-oxygenator
